# Expasy, the Swiss Bioinformatics Resource Portal, as designed by its users

**DOI:** 10.1093/nar/gkab225

**Published:** 2021-04-13

**Authors:** Séverine Duvaud, Chiara Gabella, Frédérique Lisacek, Heinz Stockinger, Vassilios Ioannidis, Christine Durinx

**Affiliations:** SIB Swiss Institute of Bioinformatics, Quartier Sorge - Bâtiment Amphipôle, CH-1015 Lausanne, Switzerland; SIB Swiss Institute of Bioinformatics, Quartier Sorge - Bâtiment Amphipôle, CH-1015 Lausanne, Switzerland; Proteome Informatics Group, SIB Swiss Institute of Bioinformatics, and Computer Science Department, University of Geneva, CH-1227 Geneva, Switzerland; Section of Biology, University of Geneva, CH-1205 Geneva, Switzerland; SIB Swiss Institute of Bioinformatics, Quartier Sorge - Bâtiment Amphipôle, CH-1015 Lausanne, Switzerland; SIB Swiss Institute of Bioinformatics, Quartier Sorge - Bâtiment Amphipôle, CH-1015 Lausanne, Switzerland; SIB Swiss Institute of Bioinformatics, Quartier Sorge - Bâtiment Amphipôle, CH-1015 Lausanne, Switzerland

## Abstract

The SIB Swiss Institute of Bioinformatics (https://www.sib.swiss) creates, maintains and disseminates a portfolio of reliable and state-of-the-art bioinformatics services and resources for the storage, analysis and interpretation of biological data. Through Expasy (https://www.expasy.org), the Swiss Bioinformatics Resource Portal, the scientific community worldwide, freely accesses more than 160 SIB resources supporting a wide range of life science and biomedical research areas. In 2020, Expasy was redesigned through a user-centric approach, known as User-Centred Design (UCD), whose aim is to create user interfaces that are easy-to-use, efficient and targeting the intended community. This approach, widely used in other fields such as marketing, e-commerce, and design of mobile applications, is still scarcely explored in bioinformatics. In total, around 50 people were actively involved, including internal stakeholders and end-users. In addition to an optimised interface that meets users' needs and expectations, the new version of Expasy provides an up-to-date and accurate description of high-quality resources based on a standardised ontology, allowing to connect functionally-related resources.

## Graphical Abstract

**Graphical Abstract:**
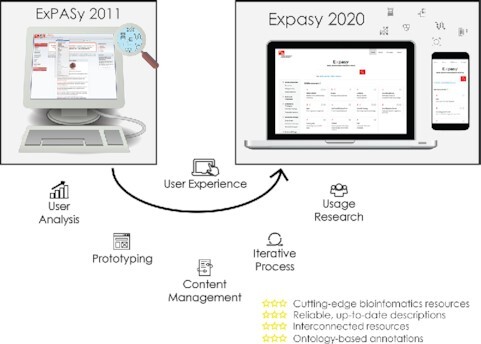
The illustration of Expasy's redesign through a user-centric approach: all the elements that contributed to the process (icons) and the main strengths (stars) of the new implementation.

## INTRODUCTION

Databases, software tools and on-line services are essential resources in the daily work of most life scientists. They are key to the long-term preservation of scientific data and the reproducibility of life science studies. As on-line bioinformatics usage spread and diversified, portals offering single access points were created (1,2) and later on, structured catalogues were proposed (3,4). In this landscape, the ExPASy portal pioneered. It was created in 1993 referred to as ‘the Expert Protein Analysis System’ with a primary focus on protein knowledge (5). It was the first life science website - and among the 150 very first websites in the world. In the past 27 years, the portal has evolved. It was redesigned several times and the penultimate version was released in 2011, when it became the ’ExPASy SIB Bioinformatics Resource Portal’, a catalogue of bioinformatics resources (6). As detailed in (7) SIB Swiss Institute of Bioinformatics federates a Swiss bioinformatics community now close to 800 scientists. In this context, hundreds of cutting-edge resources are developed and made available to the life science community. Among those, twelve key resources—the so-called SIB resources—receive specific support from the Institute and form the SIB Resource portfolio that ranges from specialised knowledge bases such as UniProtKB/Swiss-Prot (8) (part of the UniProt consortium), neXtProt (9), STRING (10) and Bgee (11), to online tools such as SWISS-MODEL (12) and SwissDrugDesign (13).

In this article, we describe the latest version of Expasy together with its redesign process that relied on rules of User-Centred Design (UCD). UCD combines User Research (UR), which addresses the question ‘Who is the target audience?’, with User Experience (UX), which addresses the question ‘How does the user interact with the application?’. Originating from the computer industry, UCD is emerging in science, as its value has been acknowledged (14,15). Expasy was improved to maintain access to SIB’s high-quality scientific resources and to offer up-to-date and accurate information on each of them, with a more efficient and easier-to-use search engine. Additionally, a networked organisation of resource descriptions was implemented, to broaden the scope of the initial search and explore the diversity of the SIB resources. The last new aspect to the portal is the involvement of resource providers in the content update of Expasy as a crucial step toward guaranteeing quality information. With this approach, the new version focuses specifically on resources developed by or in collaboration with SIB groups, in contrast with previous editions.

## OVERVIEW

### Purpose

Expasy is an extensible and integrative portal including >160 databases and software tools developed by SIB groups. It covers a wide range of fields in life sciences and biomedical research, spanning genomics, proteomics, structural biology, evolution, phylogeny, systems biology and medicinal chemistry. Expasy is an essential tool for a large variety of users, from beginners interested in discovering bioinformatics, to advanced scientists looking for specific biological answers. Table 1 illustrates three examples of typical users of the portal (so-called personas, see below) and their corresponding use-cases. Given its usefulness for the life science community, Expasy has undergone a major overhaul in 2020. The new user interface is the result of in-depth work with users to meet their needs and expectations. Its content is a long-term commitment of the resource provider community to describe each resource accurately and in a standardised manner.

**Table 1.** Examples of user-types (personas) and their use cases

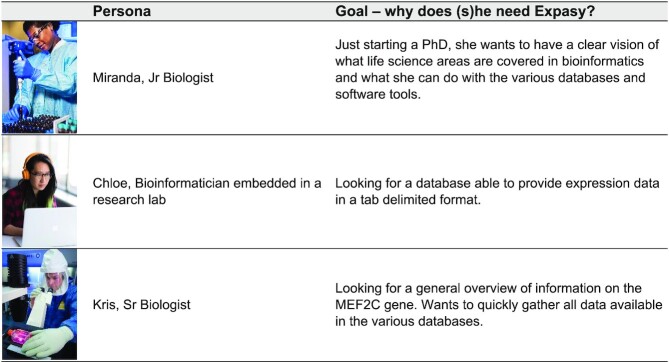

### Homepage

**Figure 1.**
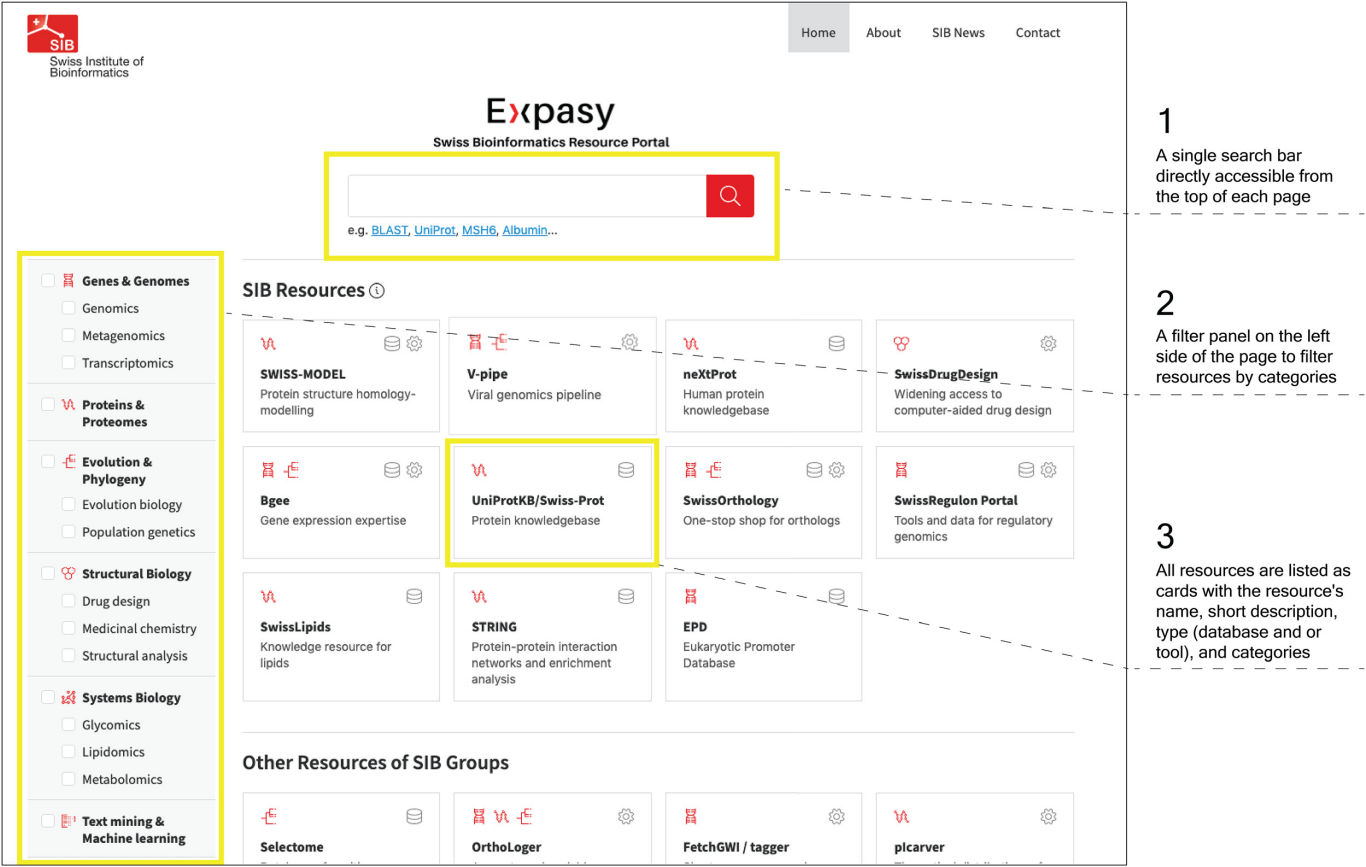
Elements on the homepage of Expasy: 1. Single search bar at the top; 2. Filter panel on the left and 3. Resources listed as cards and sorted randomly in the main section of the page.

Figure 1 shows the homepage of the new Expasy portal, including its new logo. A search bar is directly accessible from the top of each page. A filter panel is proposed on the left side of the page. It reflects the categories defined to filter the resources (see definition below). All resources are listed as cards with the resource's name, a short description, type (database and/or tool) and categories.

### Search and result page

The portal provides a single search bar allowing simultaneously two types of search: a ‘regular search’ and a ‘cross-resource search’. The results are displayed jointly on one page.

#### Regular search: browsing the resources

Expasy allows users to search for resources by name, keyword, category or description. When a user, for example, types ‘virus’ in the search bar, this results in a list of virus-related databases and software tools, such as V-pipe (16), ViralZone (17), OpenFlu (18) or COVID-19 Scenarios (19). We will refer to ‘regular search’ in the rest of the article to designate this type of search. All the resource-specific information is stored in a relational database back-end and queried using Elastic Search (https://www.elastic.co/elasticsearch/, (20)), with fuzzy search or approximate string- matching search enabled. Resources are linked one-to-the-other through ontology-based terms. As a result, they form a network of seamlessly connected resources (more details below).

#### Cross-resource search: querying the databases in parallel

In addition to the regular search, Expasy offers a ‘cross-resource search’, which allows to query a subset of web-accessible databases in parallel. Although most databases offer their own search functionality, the simultaneous query of a set of databases from a single hub is a convenient option. In this way, search results may include resources unknown to the user, yet relevant and potentially useful. This functionality, already introduced in the previous version of ExPASy (6), has been revised with the help of the respective web service providers. It now includes 19 SIB databases, such as ENZYME (21), MyHits (22), STRING(10), UniProtKB (23), ViralZone, PROSITE (24) and SWISS-MODEL Repository (see Table 2). In addition to the default behaviour (i.e., full-text search), the search engine automatically recognises certain types of formatted data, such as UniProtKB accession numbers, PDB IDs, or Ensembl IDs. As a result, it sends the query only to resources that support the specified query type. This not only optimises search time, but also provides more relevant results.

**Table 2.** Databases accessible via the cross-resource search functionality

Database name	URL	Description
SWISS-MODEL Repository	https://swissmodel.expasy.org/repository	Protein structure homology models
SwissDock	http://www.swissdock.ch/	Docking of small ligands into protein active sites
MetaNetX	https://www.metanetx.org/	Metabolic network repository & analysis
Cellosaurus	https://web.expasy.org/cellosaurus/	Knowledge resource on cell lines
Bgee	https://bgee.org/	Gene expression expertise
OMA	https://omabrowser.org/oma/home/	Orthology inference among complete genomes
Selectome	https://selectome.org/	Database of positive selection
miROrtho	http://cegg.unige.ch/mirortho	Catalogue of animal microRNA genes
OrthoDB	https://www.orthodb.org/	Ortholog evolutionary and functional annotations
neXtProt	https://www.nextprot.org/	Human protein knowledgebase
PROSITE	https://prosite.expasy.org/	Protein family and domain database
STRING	https://string-db.org/	Protein-protein interaction networks and enrichment analysis
UniProtKB	https://www.uniprot.org/	Protein sequence database
ViralZone	https://viralzone.expasy.org/	Fact sheets about viruses; linked to sequence databases.
ENZYME	https://enzyme.expasy.org/	Enzyme nomenclature database
HAMAP	https://hamap.expasy.org/	UniProtKB family classification and annotation
MyHits	https://myhits.sib.swiss/	Relationships between protein sequences and motifs
SwissLipids	https://www.swisslipids.org/#/	Knowledge resource for lipids
VenomZone	https://venomzone.expasy.org/	Portal to venom protein UniProtKB/Swiss-Prot entries

**Figure 2.**
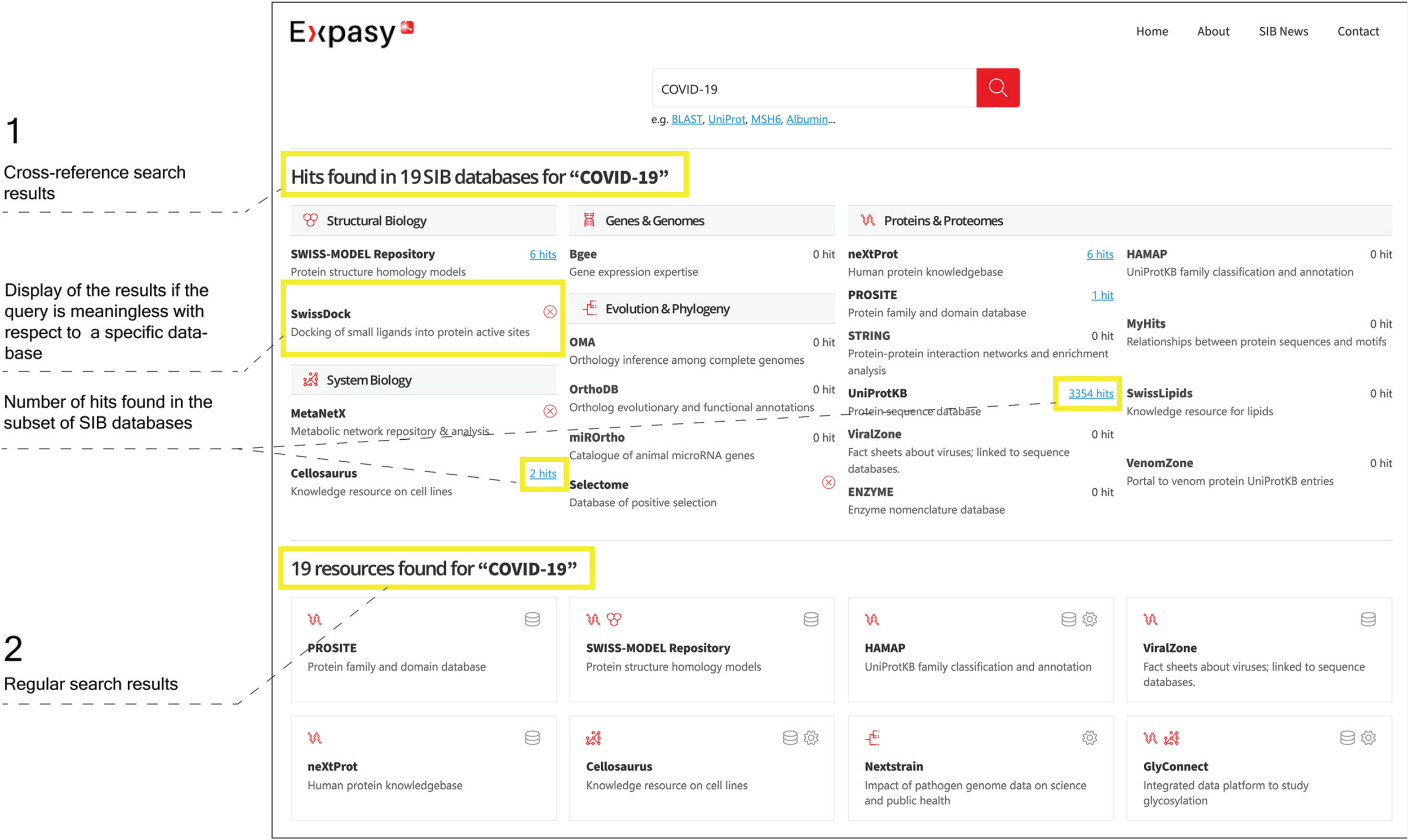
Expasy result page: the two types of search results are visually separated. 1. (Top) Cross-resource search result; with the number of hits found in each of the 19 databases; 2. (Bottom) Regular search.

Figure 2 shows the result page obtained with ‘COVID-19’ as input in the search box. This page consists of two parts:

- Top: the cross-resource search result (full-text mode)
- Bottom: the regular search result (resource names, keywords, categories and descriptions).

In the above example, the cross-resource search indicates that ‘COVID-19’ was found in two entries in Cellosaurus (25) and 3078 entries in UniProtKB. By clicking on the number of hits, the user is redirected to the query results in the respective databases. In case no result is returned (e.g. the format is not supported, or a resource server is temporarily down), an error icon replaces the number of results (see Figure 2, top). The regular search indicates that 19 resources relate to COVID-19, such as PROSITE, SWISS-MODEL Repository, ViralZone, Nextstrain (26), GlyConnect (27), SIB COVID-19 Integrated Knowledgebase (https://covid-19-sparql.expasy.org/), to name a few.

### Detailed view

Clicking on a card on the home page or on the result page shows the detailed view of the corresponding resource.

**Figure 3.**
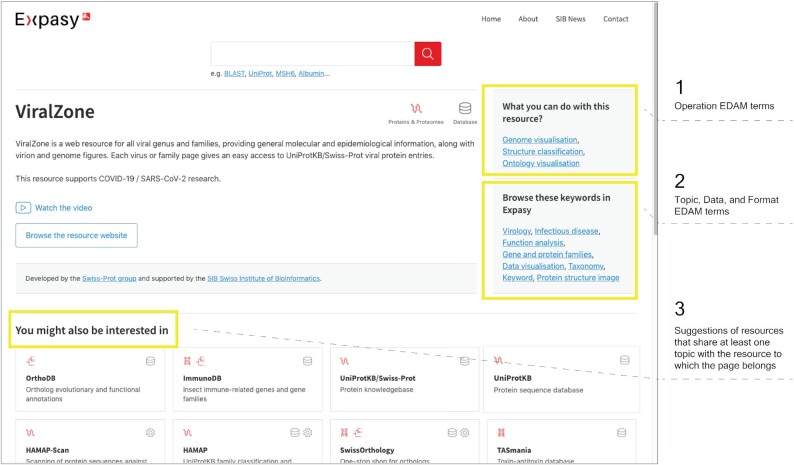
Detailed view of a resource (ViralZone). The two grey boxes display the EDAM (28) terms related to *Operations* [1] and *Topics*, *Data* and *Formats* [2]. The bottom of the page [3] lists the suggestions of resources that share at least one term of the *Topic* type with ViralZone.

Figure 3 shows a detailed view of ViralZone, a web resource for all virus genera and families. Overall, the following information describes each resource:

- Name
- Description
- Type of resource (software tool and/or database)
- Categories (Genes & Genomes, Genomics, Metagenomics, Transcriptomics, Proteins & Proteomes, Evolution & Phylogeny, Evolution biology, Population genetics, Structural Biology, Drug design, Medicinal chemistry, Structural analysis, Systems Biology, Glycomics, Lipidomics, Metabolomics or Text mining & Machine learning)
- Optional presentation video
- URL
- SIB group operating the resource
- Licence terms
- Keywords of type:

○ ‘What you can do with this resource?’
○ ‘Browse these keywords in Expasy’

- Resources of interest for the user shown as cards

Clicking on a keyword in the right box triggers a new query matching that keyword. Keywords in the old ExPASy were not standardised or following any commonly used ontology (those keywords will be designated as ‘in-house keywords’ in the rest of the article). In the new version, keywords comply with EDAM (28), a comprehensive ontology of well-established concepts in bioinformatics and computational biology. EDAM applicability to searching, categorising and automatic handling of resources has been validated by implementations in eSysbio (https://nels.bioinfo.no/), Bio-jETI (29) and EMBOSS (30), demonstrating its relevance to resource catalogues. The ontology is actively developed, maintained and supported by a team of specialists. The four sub ontologies of EDAM are used: Operation, Topic, Data and Format. In EDAM, ‘Operation’ is ‘A function that processes a set of inputs and results in a set of outputs’. Thus, all EDAM terms of the ‘Operation’ type are displayed in the box ‘What you can do with this resource?’. By clicking on one of the EDAM terms in this box, the user can retrieve all resources that perform the same task. ‘Topic’, ‘Data’ and ‘Format’ define, respectively, the domain of application, the type of information and the data format used or output by the resource. Those terms are shown in the box ‘Browse these keywords in Expasy’. The list of resources that share at least one EDAM term of the ‘Topic’ type is indicated in the ‘You might also be interested in’ section, which allows the user to explore SIB resources (see Figure 3, bottom, for details).

## THE REDESIGN PROCESS: MOTIVATION, STRATEGY AND DEVELOPMENT

**Figure 4.**
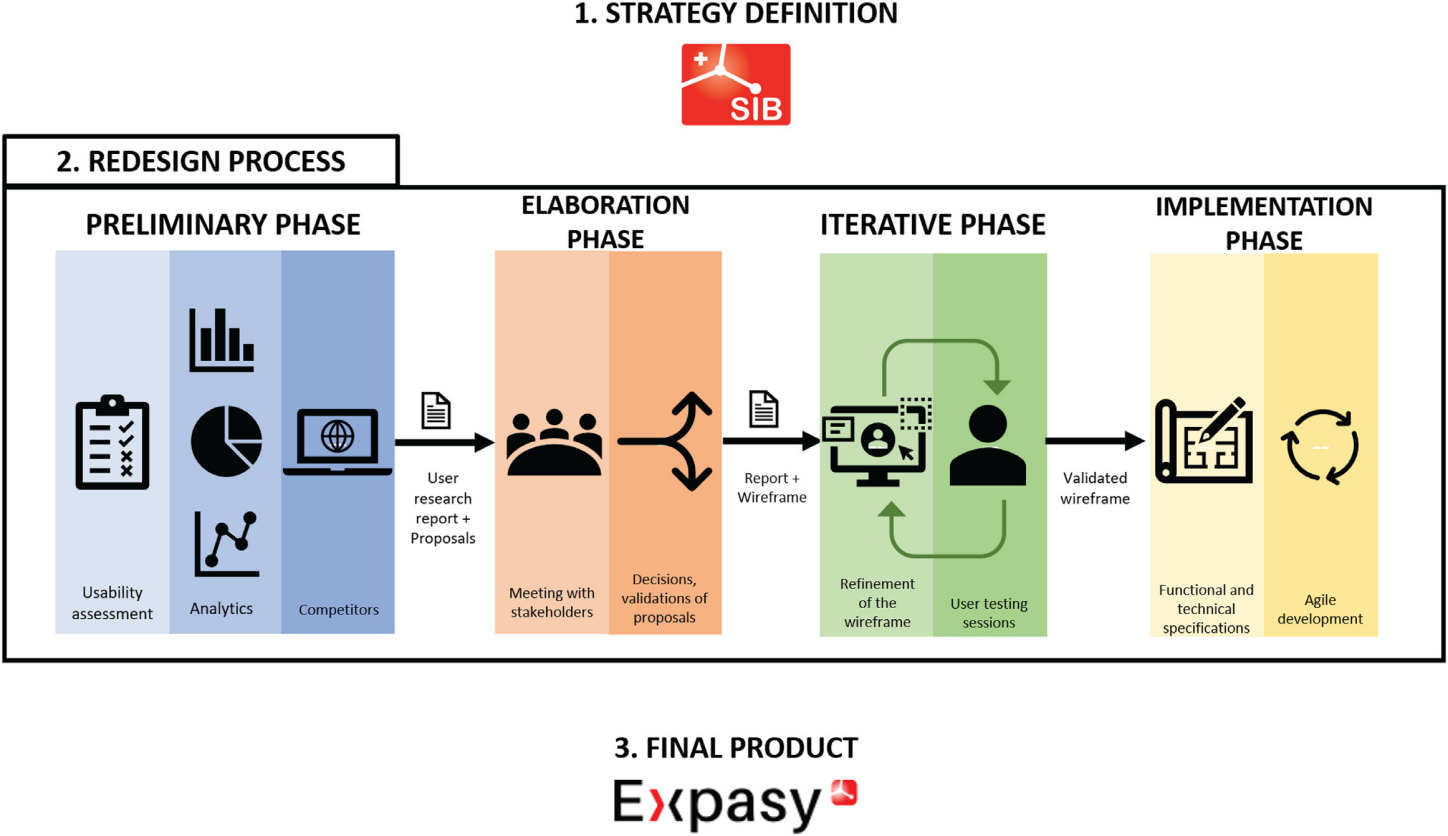
Process of development of Expasy. 1. Project strategy definition, 2. Four-phased redesign process (preliminary phase, elaboration phase, iterative phase, implementation phase) and 3. Final product.

The redesign process started in 2019. Its aim was to improve user-friendliness, visual identity and content, as well as its responsiveness on mobile devices. Additionally, we seized the opportunity to change the casing of the word ExPASy to Expasy to distinguish it from the original purpose of the website (the study of proteins) while keeping a world-renowned brand in bioinformatics. We followed a UCD approach for the redesign, anticipating that optimised user interfaces and efficient organisation of information are more conducive to scientific discoveries. Yet, professionals usually tend to wrongly assume that end-users share the same view and behave in a similar way in a given situation. In reality, the development of high scientific quality resources used to their full potential and performing adequately, must include end-users in the design process from the very beginning. With this in mind, the redesign of Expasy was carried out through four developmental phases that are described below (see Figure 4 for an illustration of the process).

### Preliminary phase

In User Experience (UX), the preliminary phase is essential for making informed decisions. To this end, we first assessed the old ExPASy according to usability, usage statistics of the website as well as user behaviour. We also evaluated a series of well-established portals in the world of life sciences, the so-called ‘competitors’.

This preliminary phase allowed us to gain significant insight into best practices and user experience improvement according to the following four criteria: usability, usage, user behaviour and benchmarking. The outcome of our evaluation is summarised in Table 3.

**Table 3.** The main outcomes and insights of the preliminary phase

Type of study		Issue	Solution/insight
**Usability**		Selection of search type (cross-resource or regular) from a dropdown menu.	Provide a single search bar for all types of search, with all types of result on a joint page.
		Website not supported by mobile devices	Make the website responsive.
**Usage**		Very limited access by mobile devices	Make the website responsive.
		Average number of acquisitions by organic searches	Improve the indexing by search engines by (i) making the website responsive; (ii) ensuring that no URL is left with a 404 (page not found) response code; and (iii) adding more content to Expasy, using controlled vocabularies (helps search engines to understand the expertise areas of Expasy).
		Low number of acquisitions by referrals	Increase the number of backlinks to Expasy from various partner websites.
**User behaviour**		Lack of navigation between resources	Ease navigation and exploration by creating a network of functionally- related resources.
**Competitor benchmarking**	EMBL-EBI and NCBI	-	Provide a single search bar (for cross-resource and regular searches). This is an example of an assumption we could validate.
	EMBL-EBI and NCBI	-	Provide a joint results page (cross-resource and regular search results). This illustrates how we took advantage of the familiarity factor.
	NCBI	-	Provide filters to narrow down the number of resources. This is an example of an assumption we could validate.
	bio.tools	-	Use an established ontology to describe the resources. This is an example of adopting best-practices.
	BioCatalogue	-	Involve the resource providers in the description of resources and the continuous revision of information. This contributed to identifying the added-value of Expasy.

#### Usability

**Figure 5.**
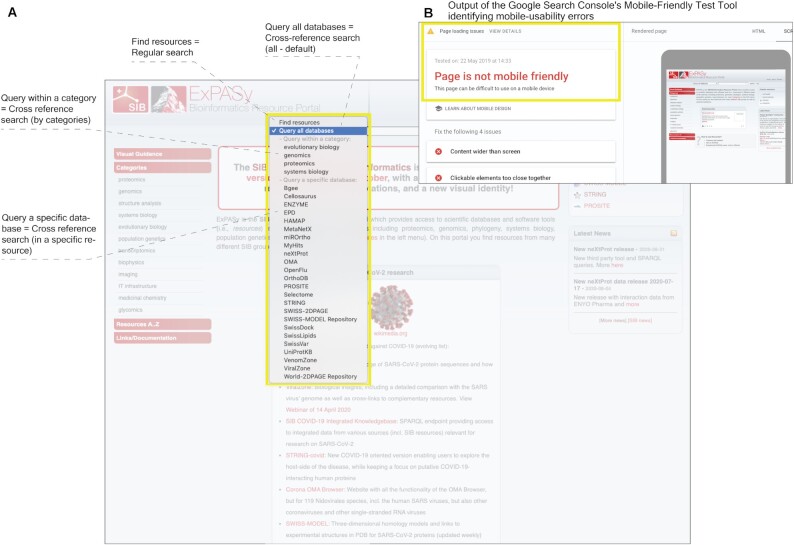
Usability issues of the previous version of ExPASy. (**A**) Dropdown menu (regular search and cross-resource search) and (**B**) output of the Google Search Console's mobile-friendly test tool.

Overall, we identified two major usability issues in the old ExPASy website (Figure 5). Firstly, users did not distinguish search types (Figure 5A). Prior to launching a search, the user was expected to select the type of search (regular or cross-resource) from a drop-down menu. Our analyses and user tests showed that very few users were aware of this option. As a consequence, users typically ran the default search (cross-resource search in 77% of cases, according to the server logs). Secondly, the website was not responsive, that is, not optimized for mobile devices (Figure 5B). Not only did this lead to very few users accessing the platform through mobile devices (around 6%), it also penalised indexing by the major search engines.

#### Usage

The study of the old ExPASy usage figures were based on Google Analytics spanning the year 2018. Overall, we observed a bounce rate of 50% which is considered as good. A bounce is when the user opens a single page on a website and exits without triggering any other action. There was an almost exclusive use of the website through desktops (94%), a huge number of acquisitions by direct access (32%), an average number of acquisitions by organic (search engine) searches (56%), and a slightly lower acquisition by referral from other, external websites (21%). Usage figures are comprehensively provided in Supplementary File S1.

#### User behaviour

A close look at the user behaviour flows revealed the low level of navigation between resources. From this observation we identified the need for further development to support navigation and exploration. Supplementary File S1 also highlights this trend.

#### Competitor benchmarking

Benchmarking is an efficient way to analyse user interfaces and community standards. In this work, we targeted well established websites to (i) evaluate ongoing practices in the field of bioinformatics, (ii) benefit from the familiarity factor, (iii) validate or disprove assumptions and (iv) assess the added value of Expasy compared to other bioinformatics portals. For this study, we focused on four portals:

1. The EMBL-EBI website (https://www.ebi.ac.uk/)
2. The NCBI website (https://www.ncbi.nlm.nih.gov/)
3. The bio.tools portal (https://bio.tools/)
4. The Biocatalogue (https://www.biocatalogue.org/)

The details of this benchmarking can be found in Supplementary File S2. Examples can be found in Table 3.

The analysis of the old ExPASy usage numbers as well as the user behaviour flows led to the creation of personas, that is, fictional characters, which are stand-ins for the different Expasy user types with characteristics, needs and expectations. Creating personas helps software developers to (i) assess the extent of different needs and expectations, and (ii) optimize the user experience (31). Three personas were created: a bioinformatician, a junior, and a senior biologist (Supplementary File S3 represents the personas and illustrates to what extend the outcome of the study supports the features of each persona).

**Figure 6.**
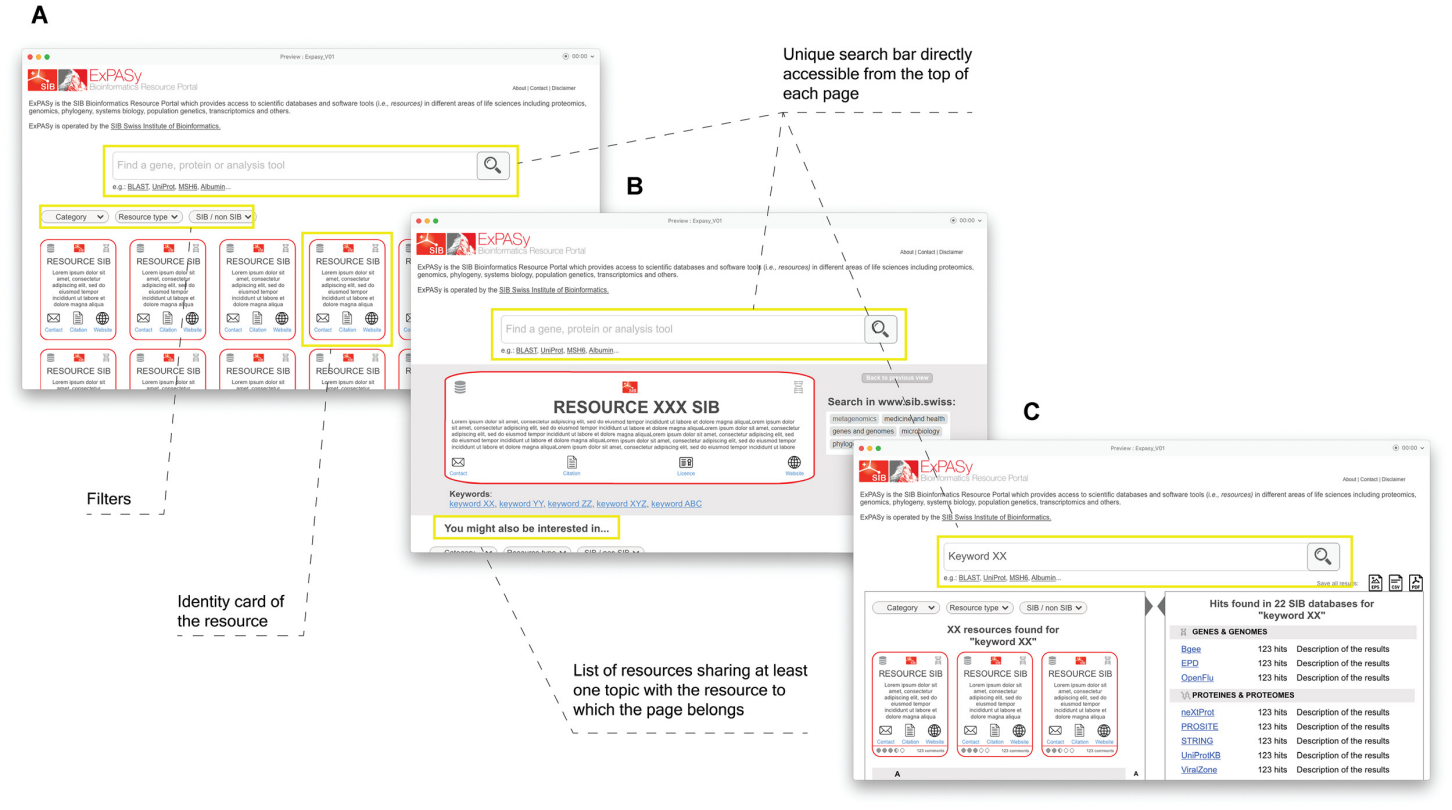
The first wireframe of the Expasy project. (**A**) Home page, (**B**) resource detailed view and (**C**) search result page.

Finally, the new Expasy was drafted as a wireframe as shown in Figure 6. A wireframe is a visual guide that represents the skeleton of a website. It depicts the layout of the user interface elements and their interactions. It lacks typographical style, colour or graphics, as the main focus is functionality, behaviour and content. In other words, it focuses on what a screen does, not on its appearance. A wireframe is employed in user tests, allowing for early feedback on products. Usually, a wireframe is iteratively enhanced at each user test to produce an optimised prototype used by developers for implementation.

The first wireframe consisted of (i) a single search bar, performing the two types of search in parallel (regular and cross-resource search) with no prior selection from a drop-down list, (ii) a homepage showing all resources listed as small cards, with a short description and additional information (website, contact, type, etc.), (iii) different types of filters (categories, resource type, etc.) to explore SIB resources according to various criteria, (iv) a detailed view of each resource providing the user with more information, and a ‘You might also be interested in…’ section allowing to broaden the scope to other SIB resources.

At this stage, the pending issues were:

- What information should the cards contain?
- What information should the detailed views contain?
- How to ensure links between resources with common features?
- Which filters should be applied?
- Which resource categories should be used to cover the diversity of SIB resources?
- How to monitor revision and long-term update of resource description?

### Elaboration phase

For the elaboration phase, we organised an interactive workshop, which brought together 17 members of the SIB community, including PIs of major SIB resources, software developers, scientists, communication and training specialists. Indeed, the SIB community brings together both the developers or providers of the resources, and the Expasy end-users. Furthermore, the workshop aimed to ease the adoption of the future version of Expasy, which is crucial for the long-term revision of resource descriptions, and more generally to sustainable content quality.

Various exercises were carried out during the workshop, and their outcome was processed in order to refine the wireframe.

More specifically, the audience unanimously confirmed the following prerequisites:

- A unique search box performing the two search types (regular and cross-resource search),
- The exclusion of citations, contact and website icon in the resource detailed view, and a clear description of the resource purpose,
- The replacement of in-house keywords by EDAM terms,
- The only indispensable filter is by category.

The field of bioinformatics evolves rapidly, therefore, through a ‘card-sorting’ exercise, the categories and sub-categories were updated, as shown in Table 4. At the end of the workshop, a new version of the wireframe was released and validated by the participants. This wireframe, resulting from a consensus of the SIB community, was then proposed to a set of external users.

**Table 4.** Categories and sub-categories in Expasy

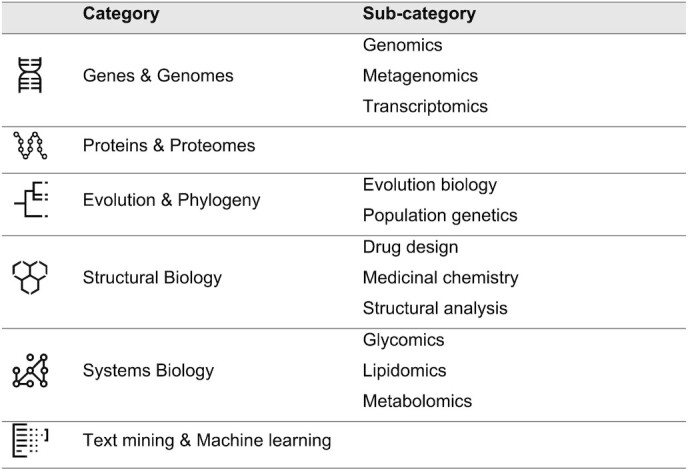

### Iterative phase

The iterative phase is about meeting the users, showing them the wireframe and improving it based on their feedback. Five users were recruited either through a mailing campaign across the authors of scientific articles citing Expasy or in person at various scientific meetings. It was established that testing a wireframe on no more than five users is sufficient to identify the major usability issues (65–85%) (32). In practise, the redundancy of user feedback is such that all important issues are brought out with the first five users and very few new ones arise when more users are consulted. The ‘law of diminishing returns’ (33) applies. Conversely, testing a wireframe on a single user is risky: a single person may perform actions by accident or in an unrepresentative manner and this may skew the test results. During the tests, users were asked to navigate the old ExPASy as well as different versions of the wireframe. Simultaneously, they were invited to compare and comment on the diverse user experiences. To avoid the anchoring bias (34), the different versions of the website were submitted in a random order. Here are some examples of exercises proposed to the users:

- Find a database collecting expert curated knowledge about glycan bindings of human pathogens
- Retrieve all the resources belonging to the ‘Evolutionary Biology’ category
- Retrieve information on the MSH6 gene. In which tissue is this gene expressed in human?

**Figure 7.**
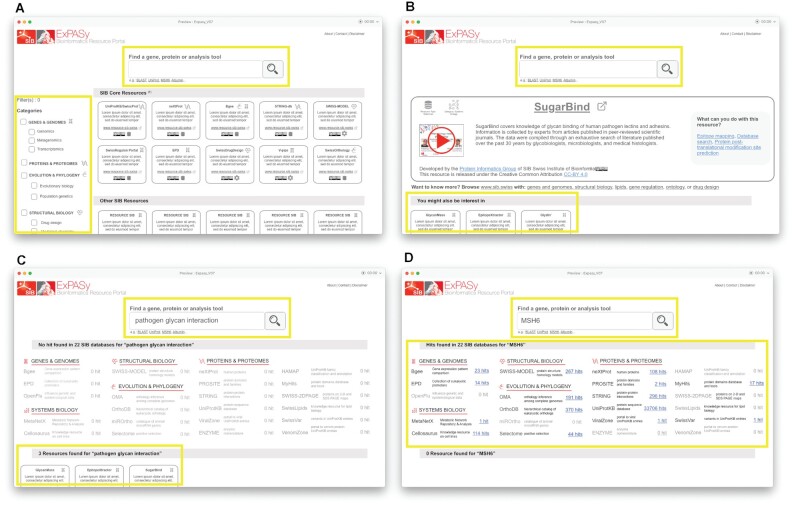
The final version of the iteratively refined wireframe as validated by the end-users. (**A**) Home page; (**B**) resource detailed view; (**C** and **D**) search result pages: C highlights on the regular search, D highlights on the cross-resource search. Note that the search bar is always present.

At the end of the iterative phase, the outcome that led to the final wireframe version (Figure 7) was the following:

- Overall, the users liked the unified search box and confirmed their unawareness of the regular search in the old implementation.
- The added value of Expasy lies in the classification of the resources by category. Besides, users preferred the panel on the left, over the initially proposed dropdown menu at the top.
- The result page, with its two types of results (Figure 7C and D), was well understood. More specifically, in the cross-resource search results, the users appreciated the classification of results by category and the short description of each queried database.

### Implementation phase

The implementation phase spans stages from the revision of the pre-existing ExPASy content to the development of the new product before going live.

To update the pre-existing data, we started by removing decommissioned resources as well as non-SIB resources. In the old ExPASy, in-house keywords were used to describe each resource main features. Manual mapping of those keywords to EDAM terms was performed and applied to each resource. The mapping result was then sent to the resource providers along with additional information such as the long and short descriptions, the contact email or the URL. Each of the resource descriptions was reviewed and enriched by the resource providers. A total of 154 resources were re-evaluated before the new Expasy implementation went live.

As for the development of the new release, a few key points had to be considered:

- Knowing that about 49% of global internet traffic can be attributed to mobile devices (source: https://gs.statcounter.com/platform-market-share/desktop-mobile-tablet/worldwide, October 2020), Expasy needed to be responsive, not only to attract more mobile device users, but also to improve indexing by the major search engines,
- Work on the Search Engine Optimization to increase the number of newcomers via organic searches,
- Update the visual identity (new stylesheet, new logo),
- Ensure the redirection of about 5000 URLs to keep existing users,
- Provide an administration interface to add/update/delete resources, open to resource providers and administrators only.

To increase speed of development and ensure user satisfaction, we opted for an agile methodology (https://agilemanifesto.org). In software development, agile refers to discovering requirements and developing solutions through a high level of engagement of cross-functional teams, enabling early delivery and continuous improvement. Eleven people (developers, business analysts, product owner, SEO expert, user interface designer, graphic designer, system administrators) participated in the implementation phase, and the new Expasy went live on 15 October 2020. The resource providers were granted access to the Expasy administration interface and provided with a video tutorial. As of January 2021, 15 new resources have been added, and 50 have been updated. Involving resource providers in the continuous content update of Expasy is crucial for ensuring accurate and relevant information. With this procedure, along with the yearly update reminder sent to resource providers, we have optimised access to up-to-date and reliable data.

## CONCLUSION

The new version of Expasy was released in October 2020. Since then, an increase of 15% in the number of daily users, compared to the same period last year, is observed. The traffic from mobile devices has increased from 6 to 10%. In the future, the SIB community will strive to provide more resources to Expasy users while ensuring that pre-existing information remains up to date. The new version of Expasy was intended as an evolving web application with a growing number of high-quality interconnected resources that forms a solid ground on which the life science community can stand. The portal is a gateway to bioinformatics, available to expert users, beginner researchers, teachers, students and more. The new version of Expasy aims to create a satisfactory user experience. Our user-centric approach was successfully applied in all phases of development and will remain our strategy in future implementations.

## DATA AVAILABILITY

Expasy is available at www.expasy.org. This website is free and open to all users and there is no login requirement.

## Supplementary Material

gkab225_Supplemental_File
