# Supplementary material for: Expasy, the Swiss Bioinformatics Resource Portal, as designed by its users
Source: Nucleic Acids Res. 2021 Apr 13;49(W1):W216–27. doi: 10.1093/nar/gkab225 (PMC8265094; doi:10.1093/nar/gkab225)
Supplement: gkab225_Supplemental_File [file gkab225_supplemental_file.pdf]

Supplementary File S1

User research (Google Analytics, year 2018)

<u>Users</u>

Parameters	Numbers
Total number	531,324
Session/user	~2
Session duration	~3 minutes
Bounce rate	~50% (very good)

<u>Geography</u>

Country	% Users
United States	22%
China	11%
India	10%
European countries	17%

<u>Devices</u>

Type	% Users
Desktop	94%
Mobile	5%
Tablet	1%

<u>Acquisition</u>

Type	% Users
Organic search	56%
Direct	32%
Referral	21%, coming from:<br>• 61% web.expasy.org<br>• 5% prosite.expasy.org<br>• 3% enzyme.expasy.org<br>• 2% NCBI

Behavior flow (all pages)

Pages	% Users
/	35%
/<category>	20%, among which:<br>• 80% /proteomics<br>• 13% /genomics<br>• 4% /transcriptomics
/<category>/<sub-category>	13%, among which:<br>• 22% /proteomics/protein_structure<br>• 20% /proteomics/protein_sequences_and_identification<br>• 13% /genomics/sequence_alignment<br>• 8% /proteomics/protein_characterisation_and_function<br>• 6% /proteomics/post-translational_modification<br>• 6% /proteomics/families__patterns_and_profiles<br>• 6% /proteomics/similarity_search_alignment<br>• 4%/proteomics/protein-protein_interaction

Behavior flow (source)

Source type	
Organic search	• 54% land on Home page<br>• 5% land on /resources/search (Home page, "Find resource" selected)<br>• 5% land on /proteomics/protein_structure<br>• 27% land on 100+ different pages.
Direct access	• 64% to the home page<br>• 12% to the /tools page<br>• 6% to the /proteomics page

Supplementary File S2

Expasy's Competitor Benchmarking

EMBL-EBI (https://www.ebi.ac.uk/)
The mission of EMBL-EBI, is to (i) make the world's public biological data freely available to the scientific community via a range of services and tools, (ii) perform basic research and (iii) provide professional training in bioinformatics. The EMBL-EBI website allows both the search for information on the institute itself (i.e. career pages, news, directory, ...) as well as the cross-resources search, and the discovery of the resources developed within the EMBL-EBI. A search bar is located at the top of the page and by default launches the 3 search types described above. A drop-down menu allows the user to refine his search by:

- Scientific search with a list of categories such as Genomic or Small molecule, which launches a cross-resources search,
- EBI content search, which allows, among other things, to discover the resources developed within the EMBL-EBI.

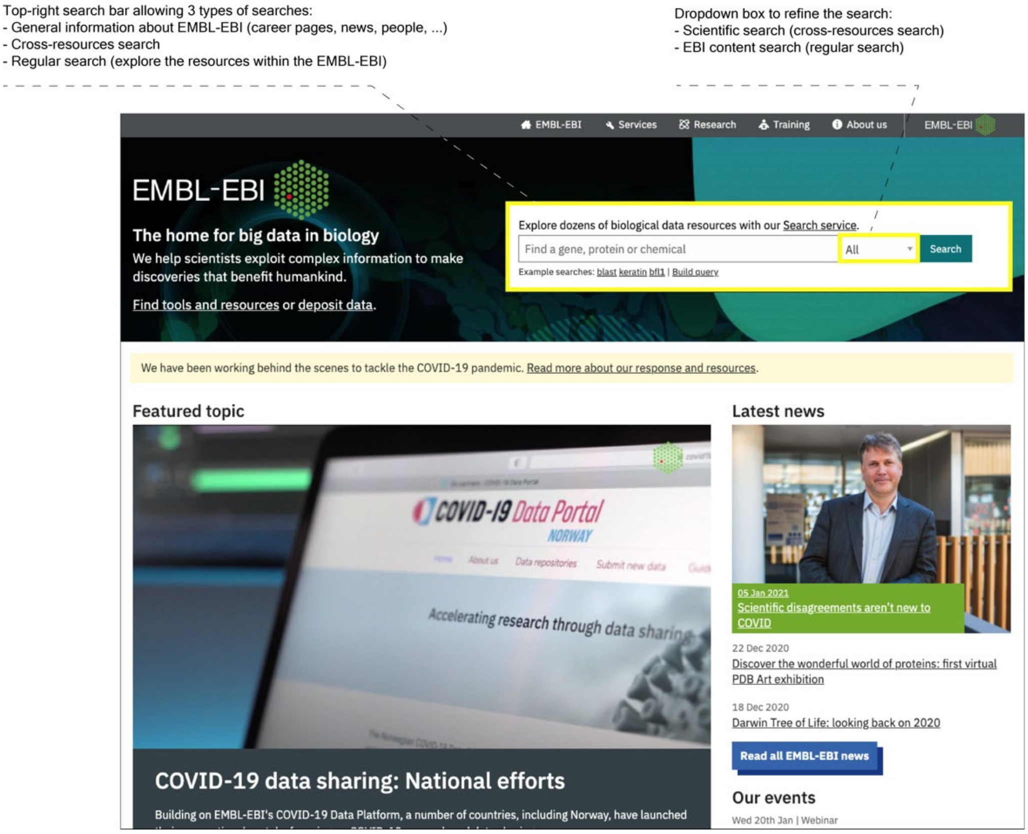

Figure 1: EMBL-EBI home page

NCBI (https://www.ncbi.nlm.nih.gov/)
NCBI's mission is, among other things, to create automated systems for the storage and analysis of knowledge in molecular biology, biochemistry and genetics and to facilitate the use of these databases and software by the research and medical community. The NCBI website includes a search bar at the top of the page. If the user wishes to limit their search to a certain dataset, they can select it from a drop-down menu. By default, the search performed is of type "cross-resources search". But in case a resource name (such as "BLAST") is detected, the corresponding web resource is described via a card at the top of

the results page. If a user is interested in exploring the resources developed within the NCBI framework, a menu on the left allows to filter the resources by category. The home page also provides a list of popular resources as well as some NCBI news.

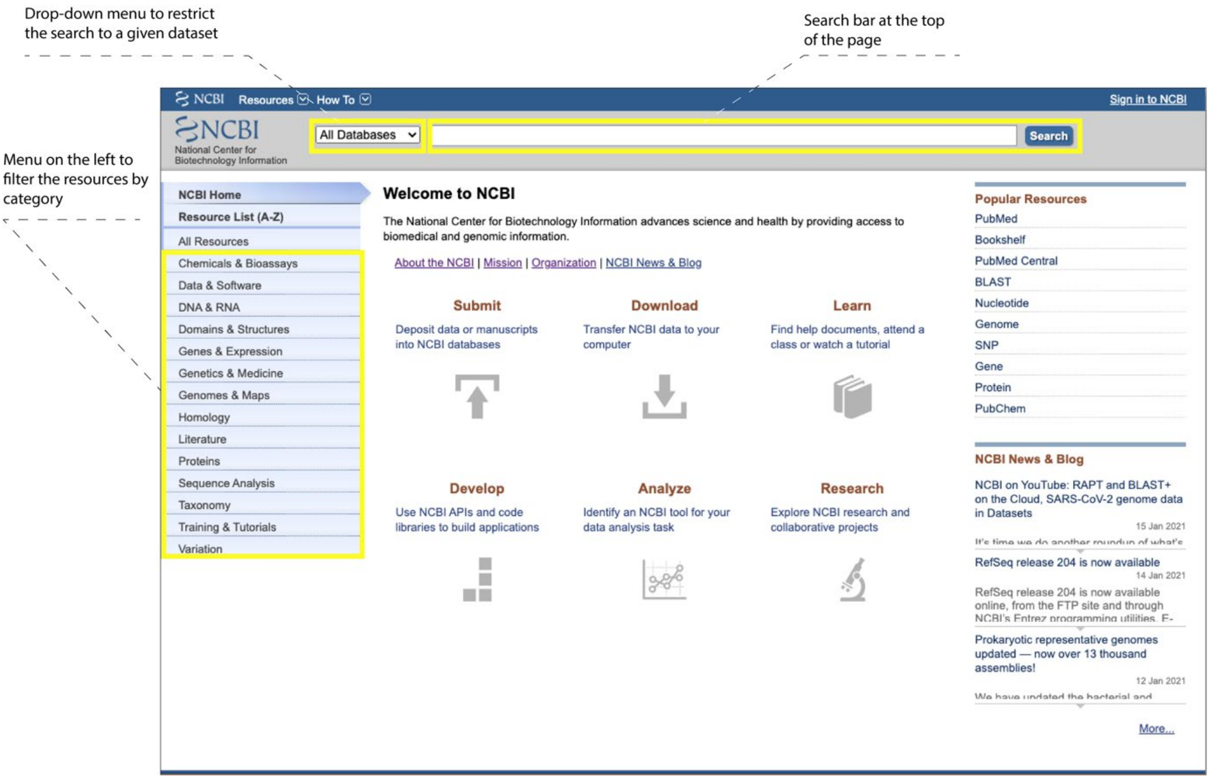

Figure 2: NCBI home page

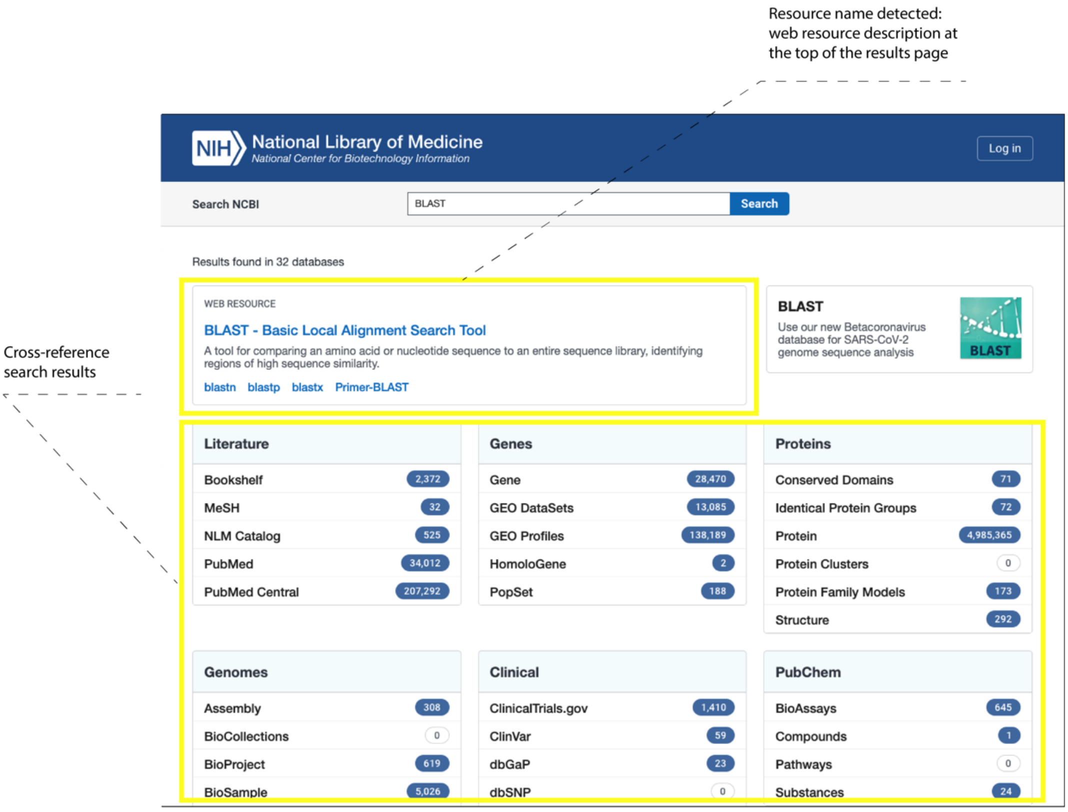

Figure 3: NCBI result page

Bio.tools (https://bio.tools/)
The bio.tools portal provides a comprehensive registry of software and databases, facilitating researchers from across the spectrum of biological and biomedical science to find, understand, utilise and cite the resources they need in their day-to-day work. A search bar allows the user to search for all information related to the resources. Through autocompletion, the user can limit the search to a certain type of information (such as Name, Topic, Operation, Input, Output, to name a few). In bio.tools, the resources are described using EDAM terms. All terms are clickable in the resource entries, allowing the user to find more resources with a given term. The resource curation is community driven and is based on more than 1000 contributors. As of January 2021, 16570 resources were described in bio.tools.

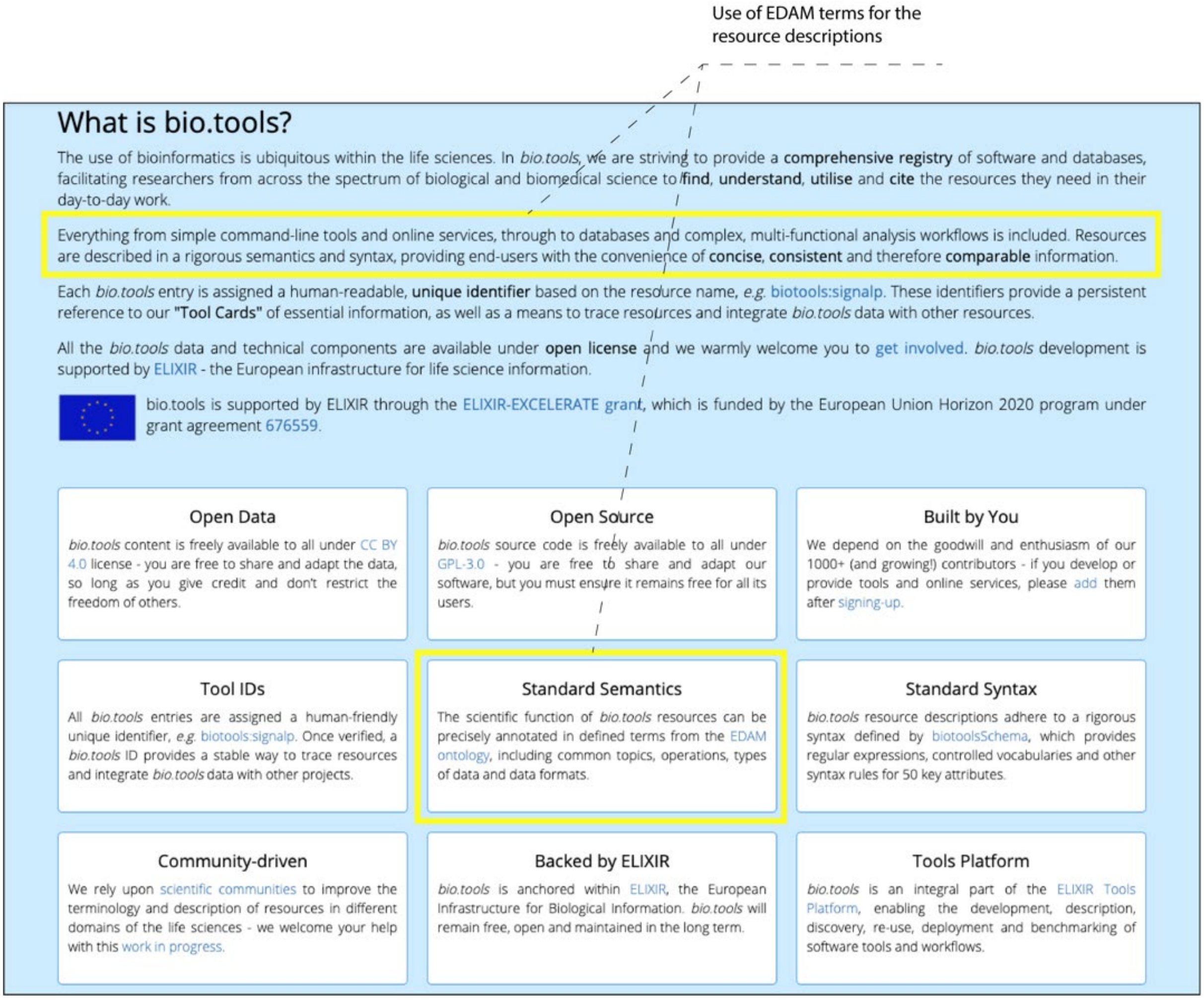

Figure 4: Bio-tools home page

The BioCatalogue (https://www.biocatalogue.org/)
The BioCatalogue is a curated catalogue of life science web services. The BioCatalogue website is freely accessible to the world thanks to a powerful search engine. Expert curators and users join forces to provide high quality annotations for services. A search bar is located at the top of the page, together with a link which leads to a list of all the BioCatalogue web services, which can later on be filtered by category thanks to a menu on the left. As of January 2021, 1431 web services were described in the BioCatalogue.

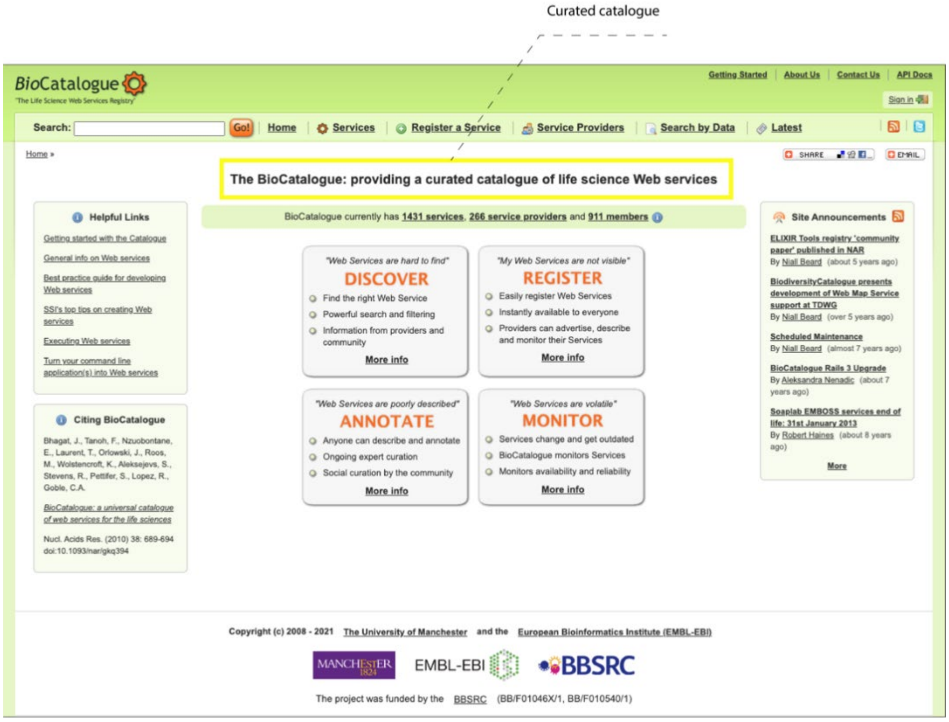

Figure 5: Biocatalogue home page

The strengths of each of the four competitors are shown in Table 1

Table 1: Good practices found in the four Expasy's competitors

Website	Strengths
**EMBL-EBI**	Unique search bar for different types of searches (institutional information, cross-reference and regular search)
**NCBI**	Unique search bar (cross-reference and regular search)<br>Filter by categories on the left<br>Joint results page (cross-reference and regular search)
**Bio.tools**	Use of EDAM terms for the resource description
**Biocatalogue**	Expert-curated catalogue

Supplementary File S3

Personas

Miranda, Jr Biologist - Beginner

After a PhD in Biology, Miranda is now moving to bioinformatics within SIB.

GOAL

- Miranda would like to explore the existing areas in bioinformatics, as well as the different tools, and databases in life sciences.
- Her fields of expertise are Glycomics and Evolutionary Biology.

PAIN POINTS

- Doesn't know what the categories are, and which resources to pick up.
- Why are some of the resources in frame?
- What are the SIB resources? Are these of good quality?
- Why are there external resources? Are these of good quality?

INTERACTION

- Miranda directly accesses https://www.expasy.org, because the site primarily showcases the SIB resources.

OPPORTUNITIES

- Add descriptions to categories.
- Better distinction between SIB resources and affiliated resources.
- Hide external resources, unless they were approved by stakeholders.
- Clean the resources in the existing database.

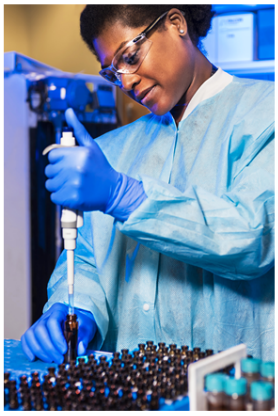

Figure 1: Miranda, Jr Biologist – Beginner. Photo credits: unsplash.com

Chloe, Bioinformatician - Intermediate

Interested in Gene Expression in C. elegans.

GOAL

- Chloe would like to find a tab-delimited file with expression data from her favorite organism. She uses her computer at work to achieve this mission.

PAIN POINTS

- Didn't notice that she had to select "Find resource" in the drop-down box.

INTERACTION

- Chloe types "Portal bioinformatics resources" in Google, and clicks on the first hit https://www.expasy.org/ ;
- Chloe types "Gene expression" in the "Query all databases" box, and clicks on the "Search" button;
- Chloe does not understand the results, and looks at the left-menu;
- Chloe clicks on "Transcriptomics", and looks at the "Databases" column;
- Chloe sees "Bgee", and clicks on "More";
- Bgee seems to fulfill her expectations, she clicks on the link.

OPPORTUNITIES

- Better indexing by search engines.
- One search box, for "Find resource" and "Query all databases".

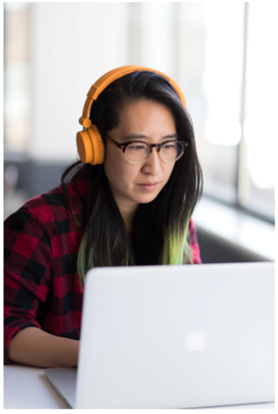

Figure 2: Chloe, Bioinformatician - Intermediate Photo credits: unsplash.com

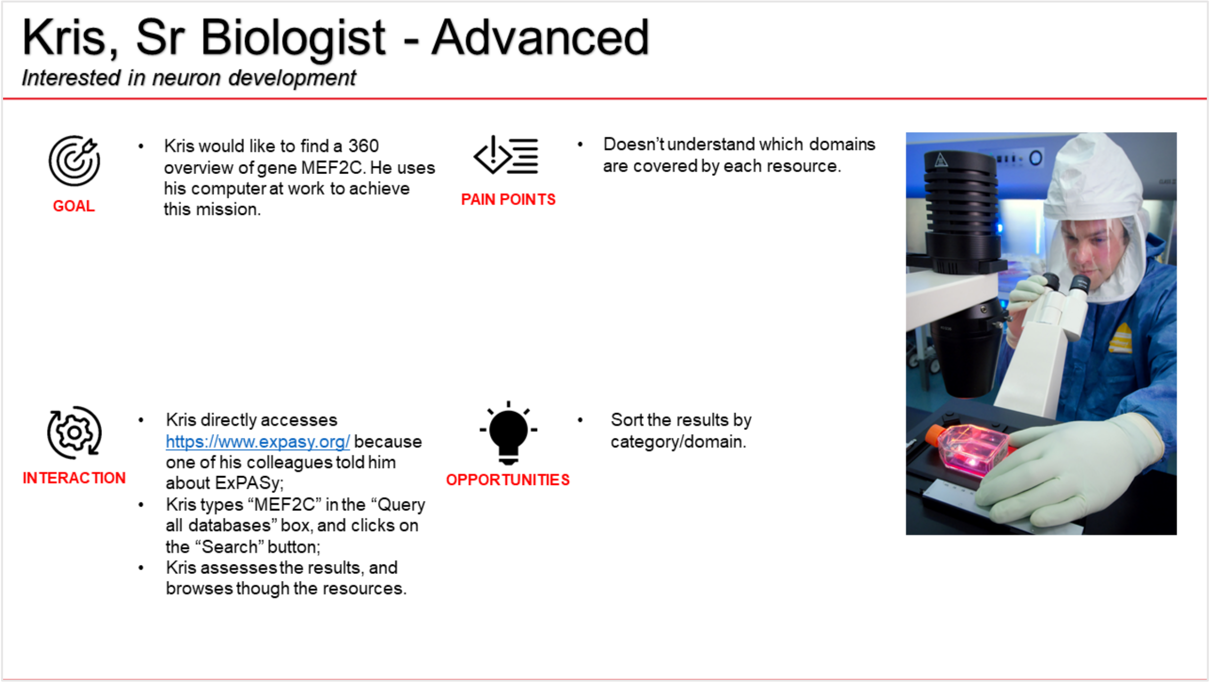

Figure 3: Kris, Sr Biologist – Advanced Photo credits: unsplash.com

Table 1: Personas are built based on the insights from the preliminary user research. The table describes how the data from Google Analytics study are reflected in the personas' characteristics and/or behaviours.

Insights from user research (Google Analytics)	Personas' characteristics and/or behaviours	
Acquisition: Direct (32%)	**Miranda**	Accesses the website directly
Devices: Desktop (94%)	**Chloe**	Uses a Desktop
Acquisition: Organic search (56%)		Uses Google
User behaviour: 54% of Google users land on the home page		Lands on the home page
User behaviour: 20% of Google users click on a category		Clicks on Transcriptomics
Acquisition: Direct (32%)	**Kris**	Accesses the website directly
User behaviour: 15% of direct accesses use the search functionality		Types a search term in the search box
